# The HLA-B*35 allele modulates ER stress, inflammation and proliferation in PBMCs from Limited Cutaneous Systemic Sclerosis patients

**DOI:** 10.1186/s13075-015-0881-1

**Published:** 2015-12-16

**Authors:** Stefania Lenna, Shervin Assassi, G. Alessandra Farina, Julio C. Mantero, Raffaella Scorza, Robert Lafyatis, Harrison W. Farber, Maria Trojanowska

**Affiliations:** Arthritis Center, Boston University School of Medicine, 72 East Concord Street, E-5, Boston, MA 02118 USA; Division of Rheumatology, University of Texas Health Science Center at Houston, Houston, TX 77030 USA; Referral Center for Systemic Autoimmune Diseases, Fondazione IRCCS Ca’ Granda Ospedale Maggiore Policlinico and University of Milan, Milan, 20122 Italy; Pulmonary Center, Boston University School of Medicine, Boston, MA 02118 USA; University of Pittsburgh Medical Center, Pittsburgh, PA 15213 USA

**Keywords:** HLA-B*35, ER stress, Inflammation, Proliferation, Scleroderma, PBMCs

## Abstract

**Introduction:**

HLA-B*35 is associated with increased risk of developing pulmonary hypertension in SSc patients. We previously reported that HLA-B*35 induces endothelial cell dysfunction via activation of ER stress/UPR and upregulation of the inflammatory response. Because PBMCs from lcSSc-PAH patients are also characterized by activation of ER stress/UPR and inflammation, the goal of this study was to assess whether the presence of HLA-B*35 contributes to those characteristics.

**Methods:**

PBMCs were purified from healthy controls (n = 49 HC) and lcSSc patients, (n = 44 with PAH, n = 53 without PAH). PBMCs from each group were stratified for the presence of HLA-B*35. Global changes in gene expression in response to HLA-B*35, HLA-B*8 or empty lentivirus were investigated by microarray analysis in HC PBMCs. Total RNA was extracted and qPCR was performed to measure gene expression.

**Results:**

ER stress markers, in particular the chaperones BiP and DNAJB1 were significantly elevated in PBMC samples carrying the HLA-B*35 allele. IL-6 expression was also significantly increased in HLA-B*35 lcSSc PBMCs and positively correlated with ER stress markers. Likewise, HMGB1 was increased in HLA-B*35-positive lcSSc PBMCs. Global gene expression analysis was used to further probe the role of HLA-B*35. Among genes downregulated by HLA-B*35 lentivirus were genes related to complement (C1QB, C1QC), cell cycle (CDNK1A) and apoptosis (Bax, Gadd45). Interestingly, complement genes (C1QC and C1QB) showed elevated expression in lcSSc without PAH, but were expressed at the low levels in lcSSc-PAH. The presence of HLA-B*35 correlated with the decreased expression of the complement genes. Furthermore, HLA-B*35 correlated with decreased expression of cyclin inhibitors (p21, p57) and pro-apoptotic genes (Bax, Gadd45) in lcSSc B35 subjects. FYN, a tyrosine kinase involved in proliferation of immune cells, was among the genes that were positively regulated by HLA-B*35. HLA-B*35 correlated with increased levels of FYN in lcSSc PBMCs.

**Conclusions:**

Our study demonstrates that HLA-B*35 contributes to the dysregulated expression of selected ER stress, inflammation and proliferation related genes in lcSSc patient PBMCs, as well as healthy individuals, thus supporting a pathogenic role of HLA-B*35 in the development of PAH in SSc patients.

**Electronic supplementary material:**

The online version of this article (doi:10.1186/s13075-015-0881-1) contains supplementary material, which is available to authorized users.

## Introduction

The contribution of genetic factors to the development of systemic sclerosis (SSc, Scleroderma) is well documented [1]. Genetic studies showed a higher incidence of SSc in families with a history of disease compared to the general population (1.5–1.7 % vs 0.026 %). Also, family studies revealed that the relative risk of developing SSc in first-degree relatives of affected individuals is higher than in third-degree relatives [2]. The susceptibility loci within the MHC (major histocompatibility complex) region consistently showed strong association with SSc in different cohorts and were confirmed in a large-scale genome-wide association study (GWAS) [3]. Of particular interest is HLA-B*35 (human leukocyte antigen class B), which was shown to be associated with increased risk for developing pulmonary arterial hypertension (PAH) in Italian SSc patients [4, 5]. This was confirmed in a study of Brazilian SSc patients [6]. HLAB*35 was also associated with SSc in a Choctaw Indian tribe with increased prevalence of SSc [7]. Furthermore, the association between HLA-B*35 and various other disorders as well as severe viral infections has been reported [6–9]. In particular, studies in patients with HIV (human immunodeficiency virus) infection from different geographical areas have shown a correlation between HLA-B*35 phenotype and progression of AIDS (Acquired Immune Deficiency Syndrome) [10–12].

We have previously observed that the presence of HLA-B*35 contributes to endothelial cell dysfunction by significantly increasing production of endothelin-1 (ET-1) and significantly decreasing endothelial nitric oxide synthase (eNOS) in conjunction with the upregulation of endoplasmic reticulum (ER) stress and unfolded protein response (UPR) in cultured endothelial cells (ECs) [13, 14]. Furthermore, HLA-B*35-dependent activation of ER stress/UPR correlated with upregulation of the interferon-regulated genes and other inflammatory genes, including IL-6.

A subsequent study using peripheral blood mononuclear cells (PBMCs) obtained from limited cutaneous systemic sclerosis (lcSSc) patients also demonstrated elevated levels of several ER stress markers, particularly in lcSSc patients with PAH. A positive correlation between selected ER stress/UPR markers (BiP/GRP78, glucose regulated protein, and DNAJB1) and IL-6 was also observed, suggesting that ER stress/UPR may have a role in the altered function of circulating immune cells in patients with lcSSc [15].

Given the association of the HLA-B*35 with the ER stress and UPR in endothelial cells, in this study, we examined in greater detail the potential contribution of HLA-B*35 to the dysregulated pathways in lcSSc lymphocytes.

## Materials and methods

### Study participants

The study subjects consisted of the patients described in our previous study [15, 16], as well as additional healthy controls and lcSSc patients (described in Additional file 1: Table S1). The Boston University Medical Center Institutional Review Board (Boston, MA, USA) reviewed and approved the conduct of this study. Informed consent was obtained from all patients and healthy subjects. Subjects included 97 patients with lcSSc according to diagnostic [17] and subtype criteria [18] (44 with PAH and 53 without PAH), as well as 49 normal healthy controls.

Patients with lcSSc were stratified into those with or without PAH based on echocardiography or right heart catheterization (RHC); in all patients designated as PAH (n = 44), the diagnosis was confirmed by RHC (mean pulmonary arterial pressure (mPAP) ≥ 25 mm Hg, pulmonary capillary wedge pressure (PCWP) ≤15 and a pulmonary vascular resistance (PVR) ≥3 Wood units), or with PCWP > 15, but ≤ 18 considered to have PAH if adjudicated by the attending pulmonologist on the basis of PVR, PAd-PCWP gradient and trans-pulmonary gradient (and were enrolled in the REVEAL Registry as patients with PAH). Patients were considered not to have PAH if echocardiography demonstrated a pulmonary artery systolic pressure <35 mm Hg and normal right ventricular size and function. The modified Rodnan skin score (mRSS) was determined for each patient on the day of the PBMC collection [19].

SSc disease duration was measured from the onset of the first non–Raynaud’s phenomenon symptom of SSc. The mean ± SD disease duration in lcSSc patients was 10 ± 9 years. The mean ± SD age in lcSSc patients was 58 ± 9 years (80 % were women and 45 % had PAH). The mean age for the healthy controls (HCs) was 44 ± 18 years (21 of the 49 HCs were under 30 years old), and 43 % were women.

### Peripheral blood mononuclear cell isolation

Blood was collected from healthy controls and patients in CPTTM tubes designed for one-step cell separation (Becton Dickinson, Mountain View, CA, USA). The sample was then immediately mixed and centrifuged at 1,800 RCF at ambient temperature for 30 min. The PBMC cell layer was then transferred to a 15 ml tube, and PBMCs were washed twice with PBS and lysed in RNeasy RLT buffer (Qiagen, Valencia, CA, USA).

### Lentiviral infection of PBMCs

A lentiviral vector expressing HLA-B*35 (or HLA-B*8) was generated by Applied Biological Materials Inc (Richmond, BC, Canada). Briefly, the cDNA encoding HLAB*35/B*8 was cloned in the shuttle vector pLenti-II-HA-CMV, which contains a HIS tag driven by a separate CMV promoter, and was used to generate recombinant lentiviruses. Lentivirus pLenti-II-HA-CMV was used as a control vector.

Healthy control PBMCs were plated in six-well plates at a density of 0.8-1x10^6^ cells/well in RPMI supplemented with 10 % FCS and 1 % AA overnight prior to transduction. Transductions were performed using M.O.I.’s ranging from 0.1 to 1 mixing the appropriate volume of virus with 8 mg/ml Polybrene (Sigma﻿, St. Louis, MO, USA), and adding the mixture to the cells together with RPMI to achieve a total volume of 500 μL per well. After 5–6 h incubation at 37 °C an additional 500 μL of complete RPMI was added, cells were centrifuged for 30 min at 1200 rpm and culture medium was aspirated and replaced by fresh RPMI. The transduced cells were collected after 72 h. Total RNA was extracted using Qiagen’s RNeasy Mini Kits according to the manufacturer’s protocol.

### Microarray data analysis

The RNA quality and yield were assessed with an Agilent 2100 Bioanalyzer (Agilent, Santa Clara, CA, USA) and a NanoDrop Technologies ND-1000 Spectrophotometer (Thermo Fisher Scientific, Waltham, MA, USA). All microarray experiments were performed in one batch. Two hundred nanograms of total RNA were amplified and purified using a TotalPrep RNA Amplification Kit (Applied Biosystems/Ambion, Foster City, CA, USA). The amplified complementary DNA was hybridized on Illumina HT-12 arrays, and the data were extracted with Illumina Genome studio software. Pathway analysis was performed using BRB-ArrayTools (National Cancer Institute, USA). Over-represented Biocarta pathways were identified using Efron-Tibshirani’s GSA test p <0.005. Efron-Tibshirani’s test uses ‘maxmean’ statistics to identify gene sets differentially expressed. All heatmaps show unsupervised hierarchical analysis results (data have been submitted to Gene Expression Omnibus (GEO) public repository, accession number GSE73355).

### Quantitative real-time PCR

Real-time RT-PCR was performed using IQTM SYBR Green Supermix (BioRad, Waltham, MA, USA and MyiQ™ Single-Color Real-Time PCR Detection System (BioRad, Waltham, MA, USA). The amount of template used in the PCR reactions was cDNA corresponding to 200 ng reverse-transcribed total RNA. DNA polymerase was first activated at 95 °C for 3 min, denatured at 95 °C for 30 s, and annealed/extended at 61 °C for 30 s, for 40 cycles according to the manufacturer’s protocol. Expression of the housekeeping genes β-actin, GADPH, and 18S served as internal positive controls in each assay performed. After measurement of the relative fluorescence intensity for each sample, the amount of each mRNA transcript was expressed as a threshold cycle (c(t)) value. The primer sequences are available upon request.

### Statistical analysis

Comparisons of RT-PCR expression were analyzed using Mann–Whitney non-parametric analyses. Correlations were calculated using Spearman non-parametric correlations.

## Results

### The presence of HLA-B*35 allele exacerbates activation of selected ER stress/UPR genes in lcSSc PBMCs

**Fig. 1:**
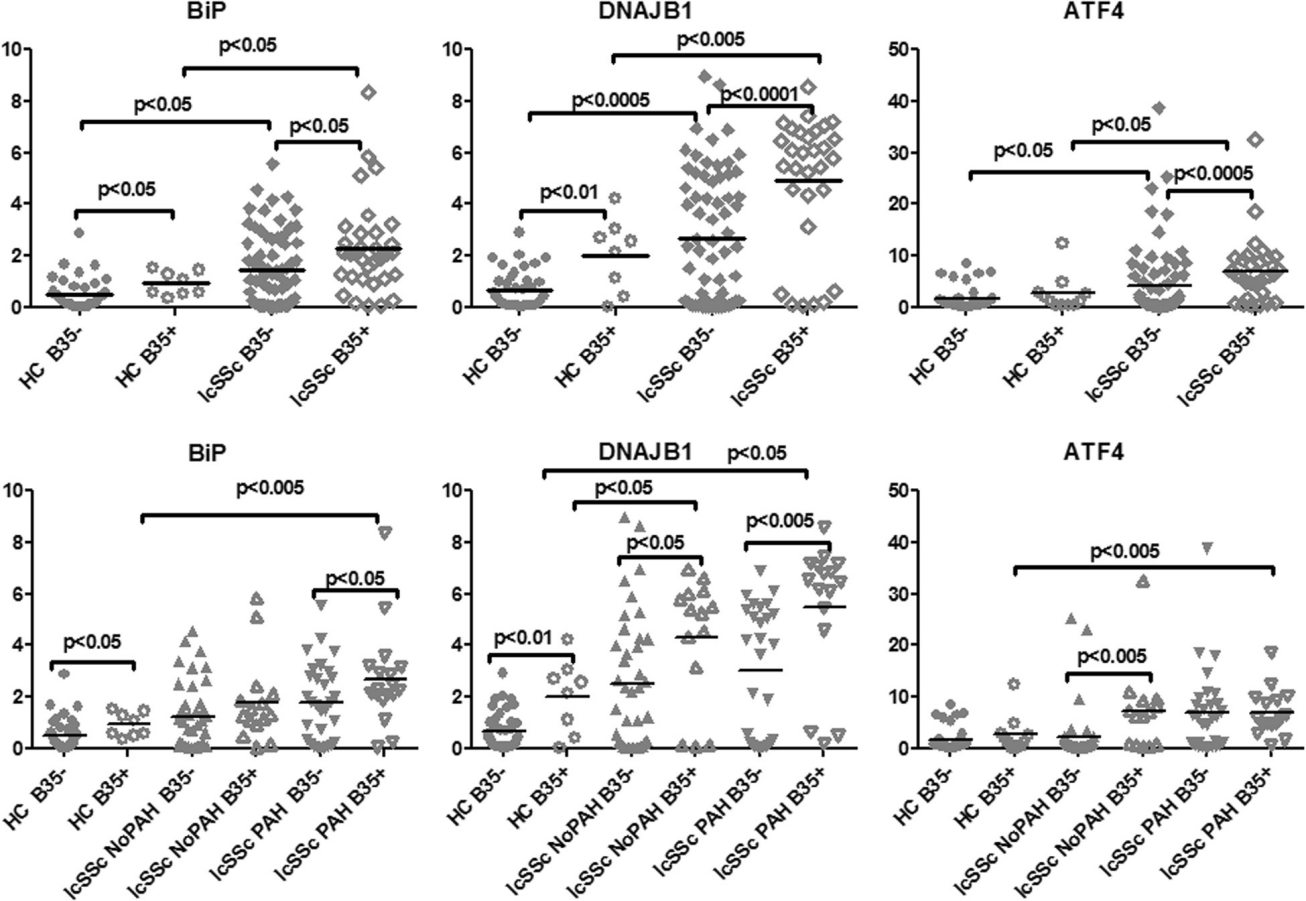
HLA-B*35 correlates with higher expression of selected ER stress/UPR genes. PBMCs were isolated from HC (n = 49), lcSSc (n = 97, NoPAH n = 53 and PAH n = 44), and grouped according to the presence of the HLA-B*35 allele: HC B35+ (n =9), HC B35- (n = 40); lcSSc B35+ (n =26), lcSSc B35- (n =71); lcSSc NoPAH B35+ (n =14), lcSSc NoPAH B35- (n = 39), lcSSc PAH B35+ (n = 12) and lcSSc PAH B35- (n = 32). mRNA levels of BiP (*left panel*), DNAJB1 (*middle panel*), and ATF4 (*right panel*) were measured by qPCR. Expression of the housekeeping genes β-actin, GADPH, and 18S served as internal positive controls. Data are expressed as the fold-change normalized to mRNA expression in a single HC sample. Each data point represents a single subject; horizontal lines show the mean. *ER* endoplasmic reticulum, *PBMCs* peripheral blood mononuclear cells, *HC* healthy controls, *lcSSc* limited cutaneous systemic sclerosis, *PAH* pulmonary arterial hypertension, *ATF4* activating transcription factor 4

The study subjects (lcSSc with PAH and without PAH), as well as healthy controls (HC) were stratified for the presence of HLA-B*35 allele (18 % of the HCs and 27 % of the lcSSc were B35-positive, of those 27 % of PAH and 26 % of NoPAH patients were B35-positive). We examined the correlation between the presence of HLA-B*35 and the expression of ER stress markers, focusing on the genes previously shown to be elevated in lcSSc-PAH PBMCs [15]. Among the previously tested ER stress markers, the chaperones BiP and DNAJB1 were consistently elevated in PBMC samples carrying the HLA-B*35 allele compared to samples negative for HLA-B*35. BiP was elevated in both B35-positive healthy controls (p < 0.05) and in B35-positive lcSSc patients (p < 0.05) (Fig. 1, upper panel). Likewise, DNAJB1 was expressed at higher levels in B35-positive HCs (p < 0.01) and B35-positive lcSSc patients (p < 0.0001). Furthermore, the highest levels of BiP and DNAJB1 were present in B35-positive lcSSc-PAH samples (BiP, p < 0.05 lcSSc-PAH B35+ vs lcSSc-PAH B35-; DNAJB1 p < 0.005 lcSSc-PAH B35+ vs lcSSc-PAH B35-) (Fig. 1, lower panel). Among the other UPR genes associated with lcSSc, only ATF4 (activating transcription factor 4) was elevated in B35-positive lcSSc vs B35-negative lcSSc (p < 0.0005), but these differences were not seen in HC samples (Fig. 1, right panel). These results suggest that the HLA-B*35 allele may primarily influence the expression of chaperons, such as BiP and DNAJ in PBMCs. Notably, a significantly increased expression of the ER stress genes was also observed in comparisons of HC B35- and lcSSc B35- samples. This suggests that besides HLA-B*35 other stressful conditions such as inflammation, infection or oxidative stress may contribute to elevated ER stress and UPR gene expression.

### Global gene expression analysis after transduction of HLA-B*35

Microarray analyses were used to further understand the role of HLA-B*35 allele. Lentivirus vector was used to ectopically express HLA-B*35 or HLA-B*8 (another antigen of class I, not known to be associated with an increased risk for developing PAH in patients with lcSSc). PBMCs were isolated from healthy controls and transduced with lentiviruses (empty lentivirus served as an additional control). The basal expression levels of a number of genes were significantly changed in response to lentivirus carrying B35 compared to B8 (or control).

**Fig. 2:**
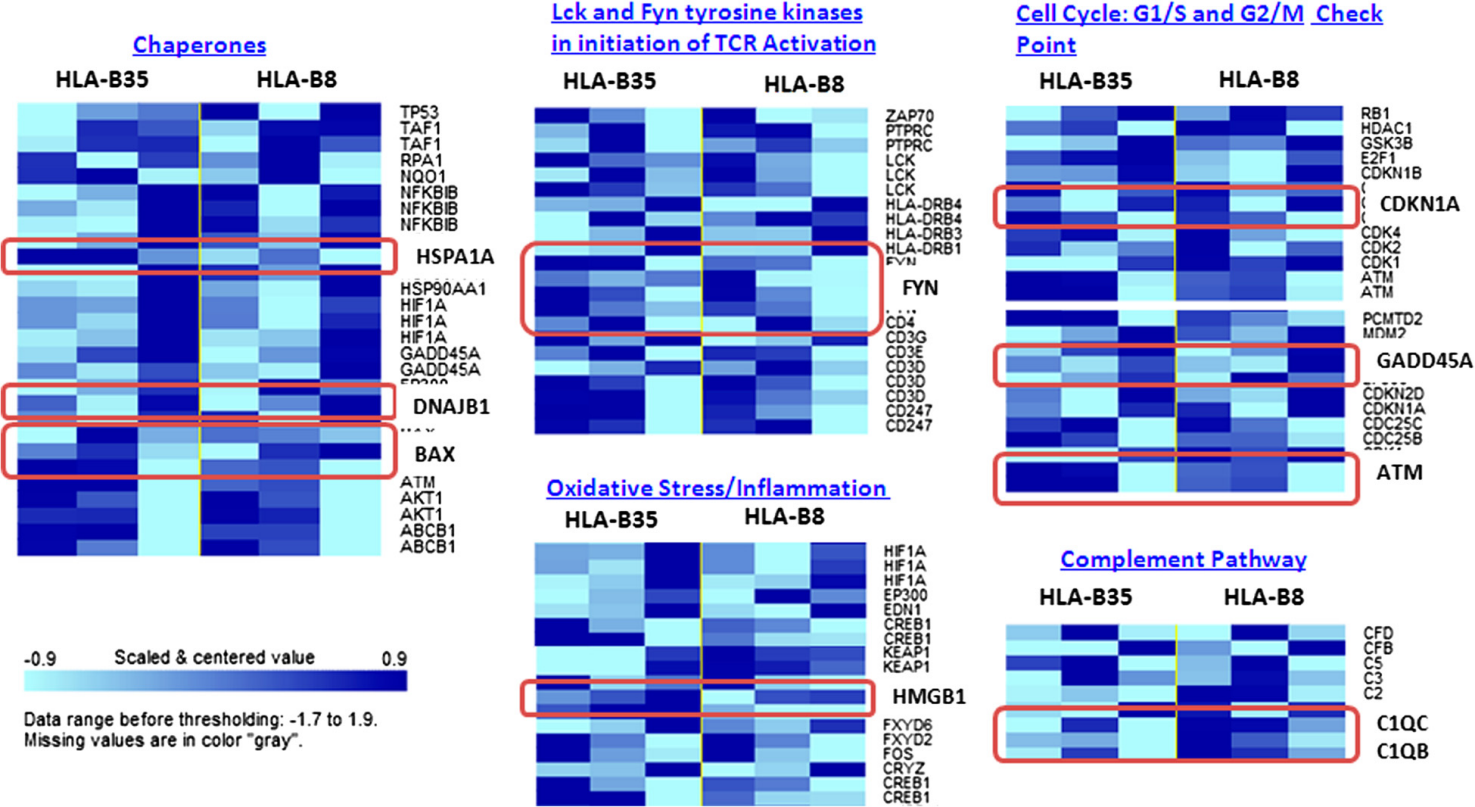
Heatmap showing the expression of gene clusters. PBMCs were isolated from healthy control and transduced with 0.1-0.5-1 MOI of lentivirus encoding HLA-B*35, HLA-B*8, or control lentivirus for 72 h. The global changes in gene expression were investigated by Illumina HT-12 arrays (Illumina Inc, San Diego, CA, USA). Among genes downregulated by HLA-B*35 lentivirus compared to HLA-B*8, we observed genes related to complement (C1QB, C1QC), cell cycle (CDNK1A), and apoptotic (Bax, Gadd45) pathways. Genes with increased expression levels were related to proliferation (FYN, ATM), inflammation (HMGB1), and ER stress/UPR (HSPA1A and DNAJB1). Expression values above the mean are indicated in dark blue, those below the mean are indicated in light blue. *PBMCs*, peripheral blood mononuclear cells, *MOI* multiplicity of infection, *ER* endoplasmic reticulum, *UPR* unfolded protein response

Sixty-four pathways were over-represented in HLA-B*35 vs. HLA-B*8 comparison (List of pathways in Additional file 2: Table S2). Among the upregulated pathways were heat shock proteins (BiP, DNAJB1), eicosanoid metabolism (ALOXA5P, arachidonate 5-lipoxygenase-activating protein), kinases (FYN, ATM), and inflammation (HMGB1, high-mobility group protein B1). Genes with decreased expression levels were related to the cell cycle pathway (inhibitor CDNK1A, cyclin-dependent kinase inhibitor 1A), the apoptotic pathway (Bax and Gadd45, growth arrest and DNA-damage-inducible 45), and the complement pathway (C1QB and C1QC, complement component 1, q subcomponent, C and B chain) (Fig. 2). Genes that showed the most pronounced changes in the array were further confirmed in PBMC cell lines isolated from four different HCs transduced with lentivirus carrying HLA-B*35 (B8 and control virus) by qPCR (Additional file 3: Figure S1). Interestingly, one of our top hits, ALOX5P, was not consistently changed in the transduced HCs used for verification and was not further investigated.

### The presence of HLA-B*35 allele in PBMCs enhances inflammation

**Fig. 3:**
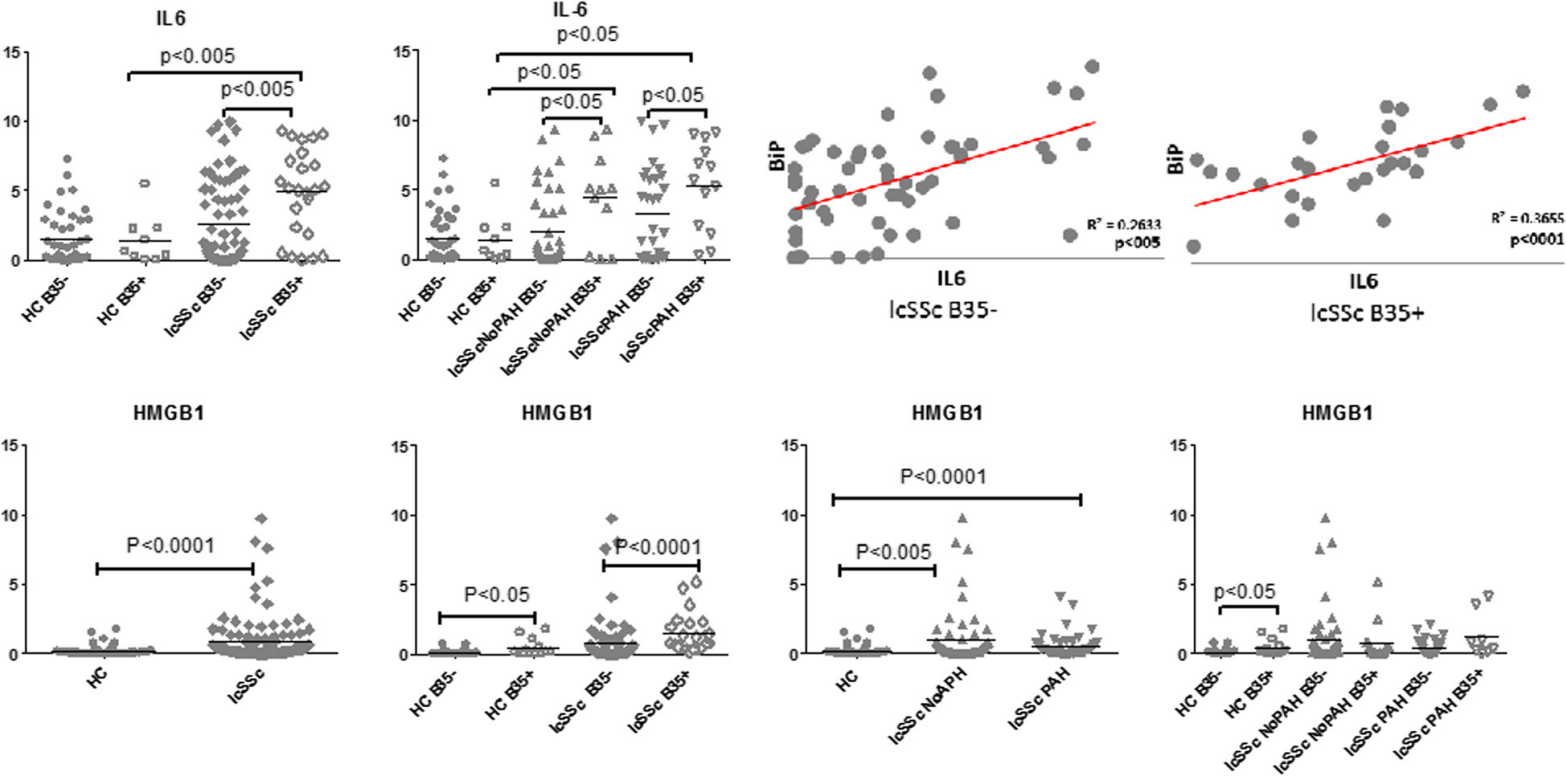
IL-6 and HMGB1 are elevated in B35-positive subjects. PBMCs were isolated from HC (n = 49), lcSSc (n = 97, NoPAH n = 53 and PAH n = 44) and grouped according to the presence of the HLA-B*35 allele: HC B35+ (n = 9), HC B35- (n = 40); lcSSc B35+ (n =26), lcSSc B35- (n =71); lcSSc NoPAH B35+ (n =14), lcSSc NoPAH B35- (n = 39), lcSSc PAH B35+ (n = 12) and lcSSc PAH B35- (n = 32). mRNA levels of IL-6 (*top panel*) and HMGB1 (*bottom panel*) were determined by qPCR. Expression of the housekeeping genes β-actin, GADPH, and 18S served as internal positive controls. Data are expressed as the fold-change normalized to mRNA expression in a single HC sample. Each data point represents a single subject; horizontal lines show the mean. *Right top* panel depicts linear regression analysis of the relationship between expression of BiP and IL-6 in PBMCs from lcSSc B35-negative and B35-positive patients. *IL-6* interleukin-6, *HMGB1* high-mobility group protein B1, *PBMCs*, peripheral blood mononuclear cells, *HC* healthy controls, *lcSSc* limited cutaneous systemic sclerosis, *PAH* pulmonary arterial hypertension

We have previously reported that interleukin 6 (IL-6) mRNA levels were significantly elevated in lcSSc vs healthy control PBMCs, with the highest levels in lcSSc-PAH PBMCs [15]. When HC and lcSSc PBMCs were stratified based on the presence of the HLA-B*35 allele, IL-6 was expressed at a higher level in HLA-B*35-positive PBMCs. The association between HLA-B*35 and higher IL-6 was observed in lcSSc PBMCs obtained from patients with and without PAH, but not in healthy controls (Fig. 3, upper panel). We have previously noted a positive correlation (r = 0.53, p < 0.0001) between mRNA expression of IL-6 and BiP in PBMC samples from patients with lcSSc [15]. Notably, IL-6 expression was also associated with the presence of HLA-B*35. When lcSSc PBMC samples were stratified based on the presence of HLA-B*35 allele, the correlation between IL-6 and BiP was higher in B35-positive samples compared to B35-negative samples (r = 0.36 vs r = 0.26).

The array analysis identified an injury response alarmin family member, HMGB1 upregulated in the presence of HLA-B*35. HMGB1 was elevated in lcSSc PBMCs vs HC PBMCs (p < 0.0001). Furthermore, the expression level of HMGB1 was elevated in B35-positive HC (p < 0.05) and lcSSc (p < 0.0001) samples. However, no differences were observed between lcSSc-NoPAH and lcSSc-PAH PBMCs or in the further stratification for the presence of antigen HLA-B*35 (Fig. 3, lower panel). These results suggest that HLA-B*35 may influence the expression of selected inflammatory genes.

### Complement genes are downregulated in HLA-B*35-positive lcSSc PBMCs

**Fig. 4:**
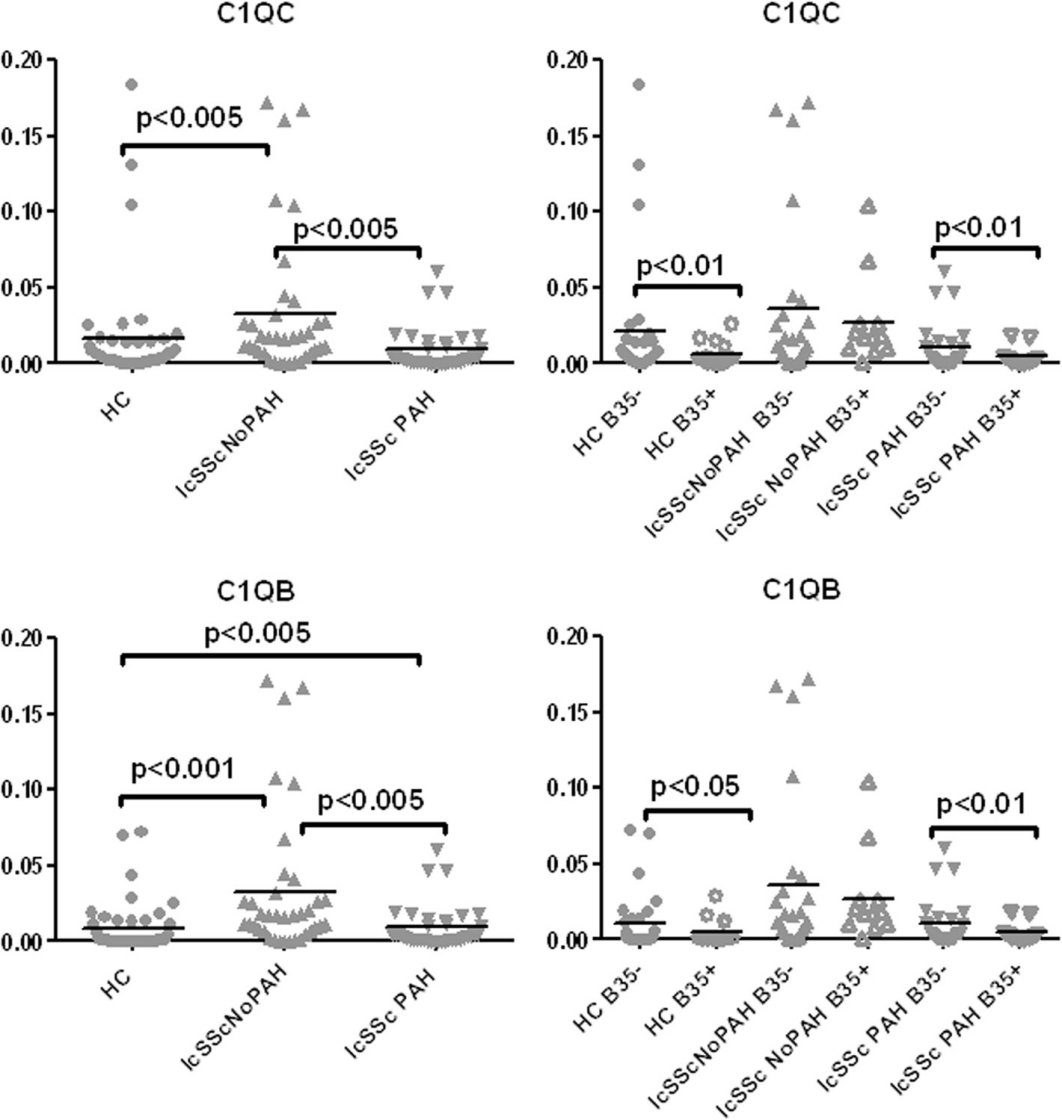
Expression of selected complement genes is decreased in HLA-B*35 positive lcSSc PBMCs. PBMCs were isolated from HC (n = 49), lcSSc (n = 82, NoPAH n = 43 and PAH n = 39) and grouped according to the presence of the HLA-B*35 allele: HC B35+ (n =9), HC B35- (n = 40); lcSSc NoPAH B35+ (n =14), lcSSc NoPAH B35- (n = 29), lcSSc PAH B35+ (n = 12) and lcSSc PAH B35- (n = 27). mRNA levels of C1QC and C1QB were measured by qPCR. Expression of the housekeeping genes β-actin, GADPH and 18S served as internal positive controls in each assay performed. *lcSSc* limited cutaneous systemic sclerosis, *PBMCs* peripheral blood mononuclear cells, *PAH* pulmonary arterial hypertension, *HC* healthy controls, *qPCR* quantitative polymerase chain reaction

Complement complexes are part of the innate immune system and their activation is known to be involved in the pathogenesis of systemic autoimmune diseases [20]. Complement genes, C1QC and C1QB, were downregulated in HC PBMCs transduced with lentivirus B35. Interestingly, both genes were elevated in PBMCs from lcSSc patients without PAH, but were expressed at significantly lower levels in lcSSc-PAH samples when compared to NoPAH samples (p < 0.005) (Fig. 4). Further stratification for the presence of B35 revealed that HLA-B*35 correlated with the low levels of the complement genes, with the lowest levels observed in B35-positive lcSSc-PAH samples (lcSSc PAH B35+ vs lcSSc PAH B35-, p < 0.01).

### HLA-B*35 correlates with low expression of cell cycle inhibitors and pro-apoptotic genes

**Fig. 5:**
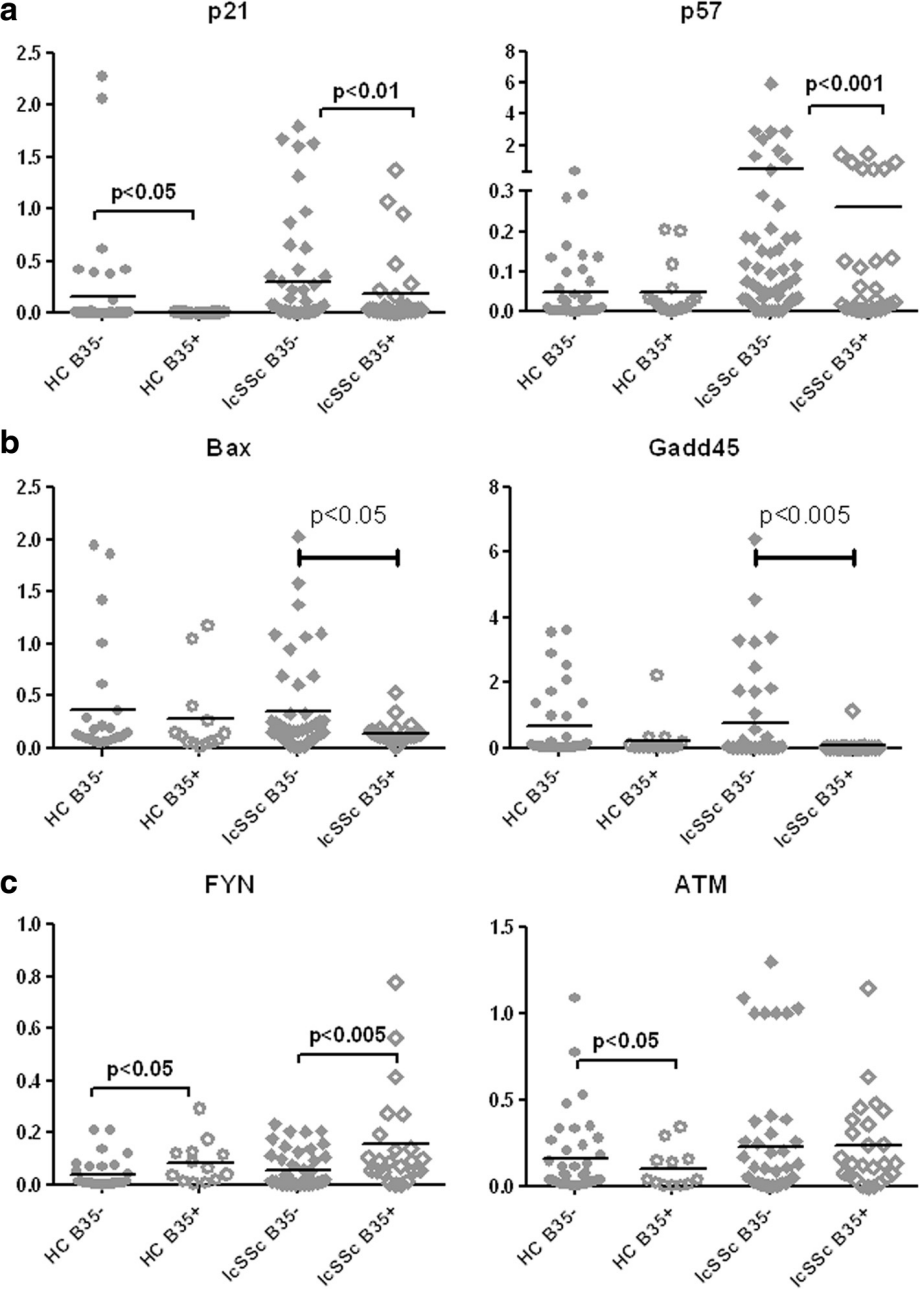
HLA-B*35 is associated with low levels of selected cyclin inhibitors and pro-apoptotic genes in lcSSc PBMCs. PBMCs were isolated from HC (n = 49), lcSSc (n = 81) and grouped according to the presence of the HLA-B*35 allele: HC B35+ (n = 9), HC B35- (n = 40); lcSSc B35+ (n = 25) and lcSSc B35- (n = 56). mRNA levels of p21, p57 **a**, Bax, Gadd45 **b** and FYN, ATM **c** were measured by qPCR. Expression of the housekeeping genes β-actin, GADPH, and 18S served as internal positive control in each assay performed. *lcSSc* limited cutaneous systemic sclerosis, *PBMCs* peripheral blood mononuclear cells, *HC* healthy controls

Healthy subject PBMCs transduced with the HLA-B*35 lentivirus showed downregulation of the genes related to growth arrest and apoptosis (p21, p57, BAX, Gadd45). Analysis of patient PBMCs also showed significantly lower levels of the cyclin-dependent kinase (CDK) inhibitors, p21 and p57, in B35-positive lcSSc PBMCs compared to B35-negative lcSSc (p < 0.01 and p < 0.001, respectively) (Fig. 5a). Healthy controls showed significantly decreased p21, but not p57, in B35-positive samples. Further stratification for the presence of HLA-B*35 in lcSSc revealed no difference in lcSSc-NoPAH B35- vs lcSSc-NoPAH B35+ while moderately lower levels were observed in lcSSc-PAH B35-positive compared to lcSSc-PAH B35-negative samples (Additional file 4: Figure S2).

Pro-apoptotic genes, such as Bax and Gadd45, were also downregulated in HLA-B*35 positive samples obtained from HC and lcSSc subjects (Fig. 5b). Low levels were also observed in B35-positive lcSSc-NoPAH and PAH samples (lcSSc PAH B35+ vs lcSSc PAH B35-, p < 0.05) (Additional file 4: Figure S2).

The above global gene expression analysis indicated that two proliferation-associated genes, FYN tyrosine kinase and ATM serine/threonine kinase, are upregulated in HLA-B*35 transduced PBMCs. Accordingly, the levels of FYN were elevated in B35-positive HCs (p < 0.05) and B35-positive lcSSc samples (*p*<0.005) (Fig. 5c). Interestingly, FYN levels were lower in lcSSc-PAH vs lcSSc-NoPAH but elevated in B35-positive subjects in both subpopulations (Additional file 4: Figure S2). In contrast to the microarray results, ATM was expressed at lower levels in B35-positive HCs, but its expression did not differ in B35-positive and B35-negative lcSSc samples (Fig. 5c). Also, no differences were observed between lcSScNoPAH and PAH, but further stratification for the presence of HLA-B*35 revealed slightly increased levels in lcSSc-NoPAH B35+ vs lcSSc-NoPAH B35- and slightly decreased levels in lcSSc-PAH B35+ vs lcSSc-NoPAH B35- (Additional file 4: Figure S2). These results suggested that the presence of HLA-B*35 may influence apoptotic and proliferative responses in PBMC subpopulations.

## Discussion

This study explored the potential contribution of HLA-B*35 to the immune dysregulation in lcSSc. An unbiased approach based on the microarrays from the human PBMCs transduced with the HLA-B*35 carrying lentivirus revealed a number of genes modulated in response to HLA-B*35. Selected genes were then verified in PBMCs obtained from patients with lcSSc as well as healthy controls. Among the genes that significantly correlated with the presence of HLA-B*35 in PBMCs were the heat shock proteins, inflammatory genes, complement genes, and genes related to cell growth and apoptosis. The upregulation of heat shock proteins typically occurs in response to various stressful conditions, including inflammation, infection, and various environmental toxins. In particular, the HSP group, which includes BiP (HSPA5) and its co-chaperone DNAJ (HSP40), is required for protein folding and is highly expressed during ER stress [21]. Notably, higher levels of heat shock proteins were also present in PBMCs obtained from healthy individuals carrying the HLA-B*35 allele, supporting the view that genetic factors could contribute to the increased levels of ER stress at least in a restricted population of SSc patients.

Inflammation and, in particular, elevated levels of IL-6 have been linked to the development of PAH [22]. Recent studies have suggested that blocking IL-6 improves both skin and interstitial lung disease in patients with dSSc (http://acrabstracts.org/abstracts/autotaxin-is-highly-expressed-in-systemic-sclerosis-ssc-skin-mediates-dermal-fibrosis-via-il-6-and-is-a-target-for-ssc-therapy/). In our study, increased levels of IL-6 in HLA-B*35-positive lcSSc PBMCs suggests that this is a genetic risk factor leading to enhanced sensitivity of HLA-B*35 leukocytes to activation. Further, our observation that the highest IL-6 levels and the highest expression of ER stress markers, BiP and HSP40, are found in B35-positive lcSSc-PAH samples, suggests that this relationship between ER stress and IL-6 plays a key role in the development of lcSSc-PAH.

Notably, we also found higher levels of HGMB1 in both HLA-B*35-positive lcSSc subjects and healthy controls. Serum levels of HGMB1 were previously shown to be elevated in SSc [23]. HMGB1, as well as HSPs, are part of the alarmin family, the endogenous molecules constitutively available and released after injury. Alarmins can promote activation of innate immune cells, recruitment and activation of antigen-presenting cells for host defense and tissue repair through activation of TLRs (Toll-like receptors) [24]. Thus, elevated HGMB1 may represent another important mediator of the effect of HLA-B*35 on immune dysregulation in lcSSc patients. Previous studies have identified altered expression levels of several additional inflammatory mediators in lcSSc PBMCs, including MCP1, IL-13, and IL-7R [25–27]. However, the presence of HLA-B35 had no effect on the expression of those genes (Lenna, unpublished results).

Among the HLA-B*35-regulated genes related to the immune system were the complement genes, C1QB and C1QC. Both genes were moderately elevated in lcSSc subjects without PAH in comparison to healthy controls; however, their expression was significantly reduced in lcSSc-PAH samples. HLA-B*35-transduced PBMCs had reduced levels of C1Q genes and this finding was verified in PBMCs from healthy controls as well as lcSSc samples with and without PAH. Complement is part of the innate immune system and its major function is to recognize and eliminate pathogens. In particular, formation of immune complexes is one of the principal ways of activating the classical pathway of the complement system. If the complement system fails in this function, waste material can accumulate and evoke an autoimmune response. Genetic deficiency of C1Q is a strong risk factor for development of SLE (systemic lupus erythematosus), triggering pro-inflammatory mediators, such as C5a and C3, and impaired cytokine production resulting in persistent and recurrent viral infections, known to be an exacerbating factor for SLE [28–31], but much less is known about the role of complement in SSc. The biological significance of the reduced levels of C1Q in carriers of the HLA-B*35 allele remains to be clarified.

Lastly, we found significantly decreased levels of selected cyclin inhibitors and pro-apoptotic genes in HLA-B*35-positive PBMCs obtained from lcSSc patients and healthy controls. On the other hand, expression of a tyrosine-protein kinase FYN was upregulated in HLA-B*35 positive PBMCs. FYN plays a role in many biological processes including regulation of cell growth and survival [32, 33]. It participates in the downstream signaling pathways that lead to T-cell differentiation and proliferation following T-cell receptor (TCR) stimulation. These results suggest that the presence of HLA-B*35 may favor proliferation of the immune cells and thus contribute to the increased inflammatory response. However, more studies are needed to fully appreciate the functional significance of the presence of HLA-B*35 allele in patients with SSc.

## Conclusions

In summary, the current study further extends our previous findings on the role of HLA-B*35 in endothelial cells [14]. In both cell types HLA-B*35 induced ER stress and inflammation related genes. Importantly, the current study verified these experimental findings in cells obtained from lcSSc patients. Notably, the presence of HLA-B*35 correlated with increased levels of alarmins, including HSPs and HMGB1, in healthy individuals, indicating that the presence of HLA-B*35 induces a stress response and is likely to sensitize endothelial and immune cells to further stressful conditions. While some of the biological consequences of HLA-B*35, including modulation of the complement and apoptotic responses, requires further investigation, this study supports the pathological role of HLA-B*35 in SSc.

## Additional files

Additional file 1: Table S1. Clinical and hemodynamic data of study subjects. PAP = pulmonary artery pressure. PCWP = pulmonary capillary wedge pressure. CO/CI = Cardiac output (L/min)/ cardiac index (L/min/m^2^). PVR = pulmonary vascular resistance. ILD = interstitial lung disease. FVC (%) = estimated forced vital capacity. DLCO = carbon monoxide diffusing capacity. SPAP = estimated systolic pulmonary artery pressure by echocardiogram. ILD was defined as present (Y = yes) or absent (N = no) based on high-resolution computed tomography assessment of the lungs. (XLS 73.5 kb) Additional file 2: Table S2. List of Gene Sets: 64 pathways sorted by LS permutation p-value. (XLS 152 kb) Additional file 3: Figure S1. Validation of array results in HC PBMCs transduced with lentivirus. Expression levels of selected genes upregulated (HSPA1A, known as BiP, DNAJB1, HMGB1, FYN, and ATM) and downregulated (CDKNA1, known as p21, Bax, Gadd45, C1QC, and C1QB) in the array analysis were verified by qPCR in four PBMC cell lines freshly isolated from healthy controls transduced with lentivirus encoding HLA-B*35 or HLA-B*8. Empty lentivirus served as additional control. Graph represents average of four different HC PBMC cell lines. (TIF 127 kb) Additional file 4: Figure S2. HLA-B*35 is associated with low levels of selected cyclin inhibitors and pro-apoptotic genes in lcSSc PBMCs. PBMCs were isolated from HC (n = 49), lcSSc (n = 81, NoPAH n = 43, and PAH n = 38) and grouped according to the presence of the HLA-B*35 allele: HC B35+ (n = 9), HC B35- (n = 40); lcSSc NoPAH B35+ (n = 14), lcSSc NoPAH B35- (n = 29), lcSSc PAH B35+ (n = 12) and lcSSc PAH B35- (n = 26). mRNA levels of p21, p57 (a), Bax, Gadd45 (b), and FYN, ATM (c) were measured by qPCR. Expression of the housekeeping genes β-actin, GADPH and 18S served as internal controls in each assay performed. (TIF 1.63 mb)

## Abbreviations

AIDS: Acquired immune deficiency syndrome
ALOXa5p: Arachidonate 5-lipoxygenase-activating protein
ATF4: activating transcription factor 4
BiP/GRP78: glucose regulated protein
C1QC/C1QB: complement component 1, q subcomponent, C / B chain
CDKN1A: Cyclin-dependent kinase inhibitor 1
EC: endothelial cells
eNOS: endothelial nitric oxide synthase
ER: endoplasmic reticulum
ET-1: endothelin-1
HC: healthy controls
HIV: human immunodeficiency virus
HLA-B*: human leukocyte antigen class B
HMGB1: high-mobility group protein B1
IL-6: interleukin 6
lcSSc: limited cutaneous systemic sclerosis
MHC: major histocompatibility complex
mPAP: mean pulmonary arterial pressure
PAH: Pulmonary arterial hypertension
PBMC: Peripheral blood mononuclear cell
PCWP: pulmonary capillary wedge pressure
PVR: pulmonary vascular resistance
SLE: systemic lupus erythematosus
SSc: systemic sclerosis, Scleroderma
TLRs: Toll-like receptors
UPR: unfolded protein response
