# Supplementary figures and images for: The HLA-B*35 allele modulates ER stress, inflammation and proliferation in PBMCs from Limited Cutaneous Systemic Sclerosis patients
Source: Arthritis Res Ther. 2015 Dec 16;17:363. doi: 10.1186/s13075-015-0881-1 (PMC4704539; doi:10.1186/s13075-015-0881-1)

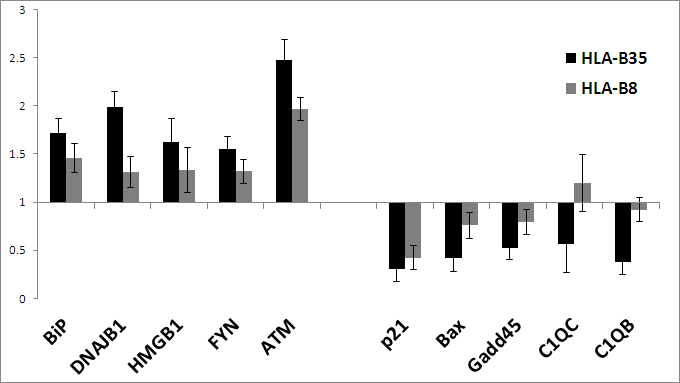

Supplement: Additional file 3: Figure S1. — Validation of array results in HC PBMCs transduced with lentivirus. Expression levels of selected genes upregulated (HSPA1A, known as BiP, DNAJB1, HMGB1, FYN, and ATM) and downregulated (CDKNA1, known as p21, Bax, Gadd45, C1QC, and C1QB) in the array analysis were verified by qPCR in four PBMC cell lines freshly isolated from healthy controls transduced with lentivirus encoding HLA-B*35 or HLA-B*8. Empty lentivirus served as additional control. Graph represents average of four different HC PBMC cell lines. (TIF 127 kb) [file 13075_2015_881_MOESM3_ESM.tif]

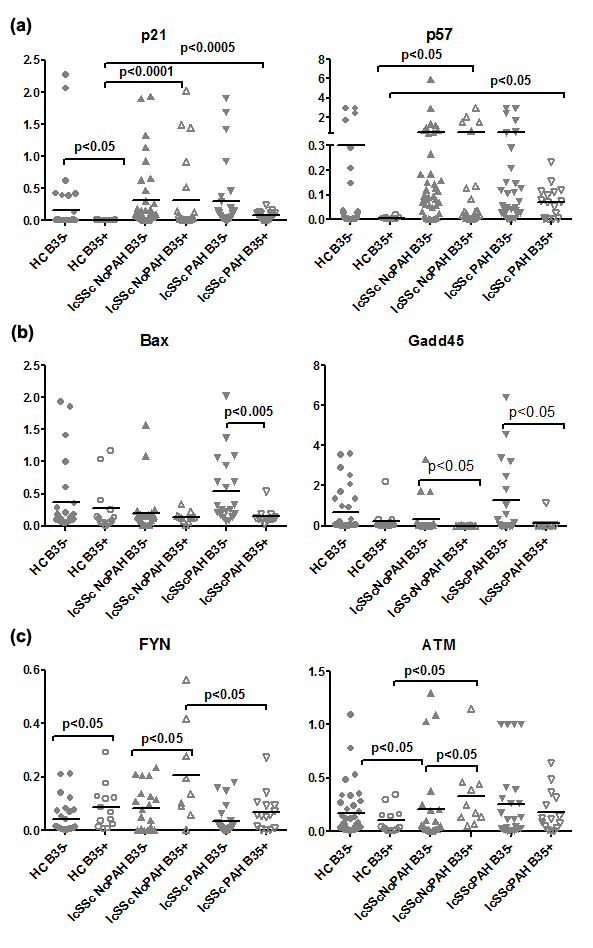

Supplement: Additional file 4: Figure S2. — HLA-B*35 is associated with low levels of selected cyclin inhibitors and pro-apoptotic genes in lcSSc PBMCs. PBMCs were isolated from HC (n = 49), lcSSc (n = 81, NoPAH n = 43, and PAH n = 38) and grouped according to the presence of the HLA-B*35 allele: HC B35+ (n = 9), HC B35- (n = 40); lcSSc NoPAH B35+ (n = 14), lcSSc NoPAH B35- (n = 29), lcSSc PAH B35+ (n = 12) and lcSSc PAH B35- (n = 26). mRNA levels of p21, p57 (a), Bax, Gadd45 (b), and FYN, ATM (c) were measured by qPCR. Expression of the housekeeping genes β-actin, GADPH and 18S served as internal controls in each assay performed. (TIF 1.63 mb) [file 13075_2015_881_MOESM4_ESM.tif]
